# Prognostic Factors of Survival in Patients With Gastric Cancer Under 45 Years Old Treated With Surgery in a Single Center in Mexico City

**DOI:** 10.7759/cureus.64183

**Published:** 2024-07-09

**Authors:** Rafael Medrano Guzman, Eulalio Jimenez Gonzalez, Atl Simon Arias Rivera, Luis E Garcia Rios, Moises Brener Chaoul

**Affiliations:** 1 Surgical Oncology, XXI Century National Medical Center, Mexico City, MEX; 2 General Surgery, Hospital Angeles Lomas, Huixquilucan, MEX; 3 Surgical Oncology, Hospital Angeles Lomas, Huixquilucan, MEX

**Keywords:** gastric cancer, surgical resection, survival, prognostic, young patients

## Abstract

Introduction

Gastric cancer is a significant major global health concern, particularly prevalent in Asia. In recent years, a large number of new cases have been diagnosed worldwide, leading to a substantial number of deaths. The disease tends to present more aggressively in these cases, leading to debates about the prognosis and survival outcomes. Nonetheless, research has shown that survival rates improve significantly when the tumor is completely surgically resected.

Materials and methods

This retrospective study included patients between 16 and 45 years old, diagnosed with gastric cancer, with the support of the pathology department, who underwent surgery in the upper GI service, in the period from January 2006 to December 2012. Data collected encompassed variables such as gender, age, tumor size, type of surgery, overall survival, disease-free period, type and histological degree of the tumor, clinical stage of the cancer, and R0 resection (curative resection).

All patients with a confirmed diagnosis of gastric cancer were included and treated with surgery and D1 limited dissection or extended D2 dissection. Patients who have received chemotherapy prior to surgical treatment and those who have been surgically treated outside the XXI Century National Medical Center were excluded.

Results

A total of 104 patients were included; the predominant histological type was diffuse adenocarcinoma accounting for 79.8% and 81.7% of the cases were histological grade 3. The most common clinical stage was IIIA in 41.3% of the cases. In 53.8% of the cases, we obtained an R0 resection. D2 lymphadenectomy was performed in 53.8% of the cases, with an overall survival rate of 82.69%.

Significant prognostic factors for survival included T4 depth with an increase in risk for mortality (OR: 25.93; 95% CI: 6.41-53.54; p=0.001), lymph node status (OR: 14.76; 95% CI: 4.6-46.83; p<0.001), and size greater than 5 cm (OR: 1.8; 95% CI: 0.61-6.35; p<0.001).

Conclusions

Gastric cancer is more common in adults aged above 60 years old, but the incidence in young adults under 45 years old has been increasing. Although young gastric cancer patients present with more aggressive tumor behavior, these patients can have similar or even better overall survival compared to older patients, being 35% in some cases, especially in the resectable setting. Further research is still needed to fully characterize the unique biology and optimal management of gastric cancer in young adults.

## Introduction

Gastric cancer represents a major global health problem especially in Asia, with around one million new cases diagnosed worldwide in 2020 with 768,000 deaths related to this diagnosis, making gastric cancer the fifth most diagnosed cancer in the world and the third cause of cancer-related deaths [1-5].

The prevalence of gastric cancer is most frequently observed in the adult population with a peak incidence around the age of 60 years. Predominating in the male sex, the incidence is considered relatively low in the population of young patients; however, the increase of this presentation has been observed for several decades. Currently, the incidence is around 2-15%.

The definition of "young patients" in gastric cancer remains debated, often categorized as individuals under 40 years old. Interestingly, this subset shows a female predominance, and the female gender has been shown to be predominant in young patients, with a male-to-female ratio of 1.0:2.5 [6,7].

Gastric cancer in young patients presents distinct clinical characteristics compared to older populations, often displaying a more aggressive course; within these characteristics, an average survival of 11-16 months is noticeable. Currently, there are different studies conducted in this group of patients, which reported a five-year overall survival of 53.6%. The main prognostic factors for survival known in this group of patients are tumor size greater than 4.5 cm, presence of lymphovascular invasion, distal location of the tumor, undifferentiated tumors or carcinoma with signet ring cells, invasion to serous, presence of distant metastasis, positive lymphatic nodes, and advanced clinical stages [8-9].

Various risk factors, mainly related to socioeconomic status, including diet, *H. pylori* infections, tobacco use, and certain occupational exposures, have been identified. A study among young Hispanic patients highlighted a high incidence of *H. pylori* seropositivity, suggesting an established risk factor for gastric cancer development [10-15].

Gastric cancer can be classified based on its anatomical site, into distal and proximal types, further categorized histologically by Lauren's classification into intestinal and diffuse. The intestinal type is most common in older patients, while the diffuse type is more frequent in younger patients. In this group of patients, it could have an increased biological aggressiveness, risk of lymphatic metastasis, higher clinical stage, and propensity to peritoneal dissemination, with a risk of recurrence of 69% [16-19].

Approximately 5-10% of gastric adenocarcinomas are thought to have a genetic basis. Most of these tumors are diffuse type, and the mutation in the germinal line is related to the gene CDH1, which codes for the cell adherence protein E-cadherin. Therefore, the identification of diffuse-type adenocarcinoma in a young patient may be strongly related to the involvement of a genetic base [20,21].

Approximately 50% of patients will present with advanced disease at the time of diagnosis; the rest will present at an early stage. The persistence of symptoms of dyspepsia for four weeks, despite adequate medical treatment and diet, entails the need to carry out a more detailed history and clinical examination and to request laboratory examinations, as well as to practice cabinet and endoscopic studies [22-29].

Several multimodal treatment schemes have been proposed, but surgery continues to be the gold standard in resectable tumors [29]. The objective of surgical treatment is to have a resection with negative margins and also a lymphadenectomy, either D1 or D2. Gastrectomy with D2 lymphadenectomy is considered the standard surgical treatment [30-32].

It has been observed that young patients, compared to older patients, in a greater proportion may be subjected to some type of surgical resection. In a study conducted on Mexican patients, up to 87% of surgical interventions were in the group of young patients compared with 66% in the older group [33].

Young patients with gastric cancer undergoing curative resection have a five-year survival rate of 78%. However, in patients who cannot be treated, a significant decrease in five-year survival was observed, 29.4%. Multiple studies have demonstrated that serous compromise is the most significant negative prognostic factor for survival, since if the neoplasm infiltrates the serous, the impact of nodular dissection on survival is reduced because surgery cannot control transcoelomic peritoneal dissemination, the leading cause of death in post-gastrectomy cancer patients [34,35].

In many cases, gastric cancer is confined to the gastric wall; however, in some patients, the tumor has invaded adjacent organs. In these cases, multiorgan resection is indicated. Surgery is the only potential curative modality for localized gastric cancer, offering the only possibility of long-term survival. Young patients undergoing curative resection of gastric cancer had a better survival rate compared to those who could not undergo surgery (average survival: 70.6 months vs. 9.1 months; p<0.001). There is currently no standard post-radical chemotherapy treatment for advanced gastric cancer; however, some meta-analyses indicate that adjuvant chemotherapies have significant survival benefits in patients with gastric cancers [34-38]. 

## Materials and methods

This retrospective study included patients aged 16-45, diagnosed with gastric cancer, who underwent surgical management in the upper GI service, in the period from January 2006 to December 2012. We collected the following variables: gender, age, tumor size, type of surgery, overall survival, disease-free period, type and histological degree of the tumor, clinical stage of the cancer, and R0 resection (curative resection).

All patients with a confirmed diagnosis of gastric cancer as a histopathological outcome were included. The patients were treated with a R0 resection with a D1 lymphadenectomy or extended D2 dissection. In some cases, it was necessary to perform a multi-structural resection. Patients with T1, T2, T3, and T4a tumors with R0 resection were included in the study. Patients who have received chemotherapy prior to surgical treatment and those who have been surgically treated outside the XXI Century National Medical Center were excluded. 

Statistical analysis 

Descriptive statistics were carried out to know the distribution of the variables; for quantitative variables, they will be determined as central trend, normal distribution (average and standard deviation), and free distribution (medians and interquartile ranges). Qualitative variables are determined as frequency and percentage. The association of nominal variables will be done with the chi-squared (X2) test, and for quantitative variables, the Student t-test will be used for independent groups. The Kaplan-Meier curve will be used for survival. A value of p<0.05 shall be taken as significant. The results were expressed using pastel charts, histograms, and bar charts. For data processing, IBM SPSS Statistics for Windows, Version 24.0 (Released 2016; IBM Corp., Armonk, New York, United States) was used.

## Results

A total of 137 patients with a diagnosis of gastric cancer were identified in the group of patients under the age of 45. One (0.7%) patient operated outside the unit, 13 (9.48%) patients with a histopathological diagnosis other than gastric cancer, 15 (10.94%) patients not having a clinical record in the unit (ARIMAC file), and four (2.9%) patients with incomplete clinical records were eliminated. Hence, 104 patients met the criteria for our study. 

The evaluable patient group consisted of 47 males and 57 females, and the age of presentation of gastric cancer was an average of 39 years (DE±5 years). Overall, 47.1% of patients presented an Eastern Cooperative Oncology Group (ECOG) of 2 at the time of the first evaluation in the external consultation. The most frequent symptoms presented were nausea and vomiting (23.1%), abdominal pain (22.1%), dyspepsia (19.2%), weight loss (15.4%), high digestive tube bleeding (11.5%), dysphagia (2.9%), malnutrition, anemia, and presence of abdominal tumor (Table 1).

**Table 1:** Demographic characteristics G1: well differentiated. G2: moderately differentiated. G3: little differentiated or undifferentiated. N: nodule stage. ECOG: functional scale grade ECOG: Eastern Cooperative Oncology Group; UGIB: upper gastrointestinal bleeding

Characteristics	Frequencies
Age	39±5
16-30 years	10 (9.6%)
31-35 years	94 (90.4%)
Gender	
Male	47 (45.2%)
Female	57 (54.8%)
ECOG	
ECOG 1	29 (27.9%)
ECOG 2	49 (47.1%)
ECOG 3	26 (25%)
Symptoms of presentation	
Dyspepsia	20 (19.2%)
Nausea and/or vomiting	24 (23.1%)
UGIB	12 (11.5%)
Weight loss	16 (15.4%)
Malnutrition	2 (1.9%)
Anemia	2 (1.9%)
Abdominal pain	23 (22.1%)
Abdominal tumor	2 (1.9%)
Dysphagia	3 (2.9%)
Total patients	104

In the clinical-pathological assessment of the disease, the diffuse histological type (79.8%) and the intestinal type (13.5%) were identified as predominant. Also, the histological degree was mostly grade 3 in 81.7% of cases. 

The majority of patients were presented with locally advanced clinical stage (IIIA) in 41.3% of the cases, with a significant percentage of positive nodules, with an average of four positive nodes (DE±2), with 28 patients (26.9%) classified as N2. 

The anatomical location of the tumor most frequently in this group of patients was in the pyloric hernia (53.8%). 

Despite the characteristics of the disease previously mentioned, remote metastatic activity was identified in 40 patients (38.5%) (Table 2). 

**Table 2:** Characteristics of the disease G1: well differentiated. G2: moderately differentiated. G3: little differentiated or undifferentiated. N: nodule stage

Histological type	
Intestinal	14 (13.5%)
Diffuse	83 (79.8%)
Indeterminate	7 (6.7%)
Histological grade	
G1	0
G2	10 (9.6%)
G3	85 (81.7%)
Not reported	9 (8.7%)
Clinical stage	
IA	2 (1.9%)
IIA	1 (1.0%)
IIB	4 (3.8%)
IIIA	43 (41.3%)
IIIB	21 (20.2%)
IIIC	23 (22.1%)
IV	10 (9.6%)
Nodal status	4 ±2
NX	46 (44.2%)
N0	5 (4.8%)
N1	17 (16.3%)
N2	28 (26.9%)
N3a	8 (7.7%)
N3b	0
Tumor location	
Cardiac	11 (10.6%)
Body	5 (4.8%)
Fundus	18 (17.3%)
Pyloric antrum	56 (53.8%)
Diffuse	14 (13.5%)
Distant metastasis	
Yes	40 (38.5%)
No	64 (61.5%)

Table 3 shows the characteristics at the time of the surgical procedure. Complete resective surgery was performed in 61 patients, obtaining R0 surgery in 53.8%; in 41.3%, the surgery could not be performed due to the criteria of irresectability, among which carcinomatosis, remote metastatic tumor activity, and tumor activity in the celiac trunk are mentioned. Of the patients who were able to undergo oncological surgery, 56 of them, surgery included D2 lymphadenectomy (53.8%), with negative margins of 56.7%. 

**Table 3:** Characteristics of the surgery performed D: type of lymphadenectomy. D1: consists of the removal of all perigastric lymph nodes. D2: includes the removal of all the perigastric arteries and the branches of the celiac trunk (hepatic artery, splenic artery, and left gastric artery)

Characteristics	Frequency
Type of surgery	
Total gastrectomy	29 (27.9%)
Subtotal gastrectomy	28 (26.9%)
Exploratory laparotomy	43 (41.3%)
Multi-structural	4 (3.8%)
Total	104
Type of lymphadenectomy	
D1 lymphadenectomy	1 (1.0%)
D2 lymphadenectomy	56 (53.8%)
None	47 (45.2%)
Surgical margins	
Positive	45 (43.3%)
Negative	59 (56.7%)
Resectable disease	
Yes	56 (53.8%)
No	48 (46.2%)

**Figure 1:**
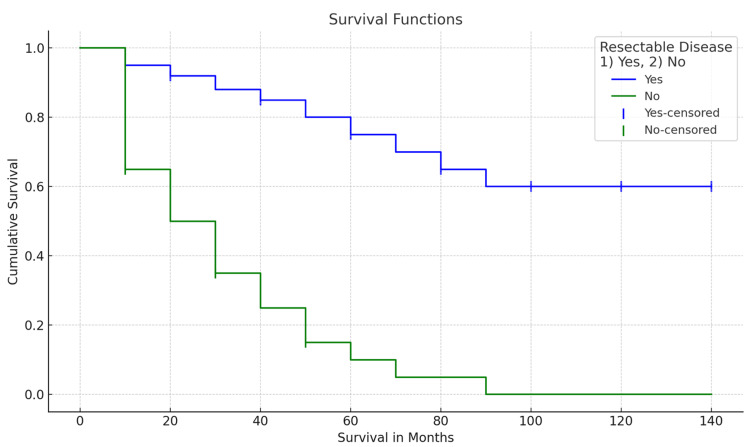
Survival

For survival, the Kaplan-Meier curve was used, with a CI of 69.057-102.24, achieving a total survival of 82.69% in the resective surgical group, with a p=0.000 value. Greater survival was observed in patients who underwent R0 resection (Table 3). According to the relationship that exists between the different variables, when performing the statistical analysis of the survival of patients in the study group, no significant difference was observed for survival according to the histological type (p=0.874). On the other hand, the value of the degree of differentiation did not show a significant difference (p=0.317), and as warned, the variables that resulted as significant prognostic factors for survival are the clinical stage, the nodular state, the presence of remote metastasis, the type of lymphadenectomy performed, and resection R0, all with a p<0.05 value (Table 4, Figure 1).

**Table 4:** Period free from disease G1: well differentiated. G2: moderately differentiated. G3: little differentiated or undifferentiated. N: nodule stage. D: type of lymphadenectomy. D1: consists of the removal of all perigastric lymph nodes. D2: includes the removal of all the perigastric arteries and the branches of the celiac trunk (hepatic artery, splenic artery, and left gastric artery)

Variable	<12 months	1-4 years	>5 years	P-value
Histological type				0.494
Intestinal	5	4	5	
Diffuse	43	17	23	
Indeterminate	5	0	2	
Degree of differentiation				0.478
G1	-	-	-	
G2	3	2	6	
G3	44	18	1	
NR	6	1	2	
Clinical stage				0.000
IA	0	1	1	
IB	-	-	-	
IIA	0	0	1	
IIB	2	0	2	
IIIA	10	9	24	
IIIB	11	8	2	
IIIC	20	3	0	
IV	10	0	0	
Nodal status				0.000
N0	0	2	3	
N1	7	4	6	
N2	4	5	19	
N3	2	4	4	
NX	-	-	-	
Distant metastasis				0.000
Yes	40	0	0	
No	13	21	30	
Type of lymphadenectomy				0.000
D1	0	1	0	
D2	12	14	30	
None	41	6	0	
Resectable disease				0.000
Yes	11	15	30	
No	42	6	9	

The predictive survival factors that increased mortality (Table 5) were T4 depth with an increase in risk for mortality (OR: 25.93; 95% CI: 6.41-53.54; p=0.001), nodular state (OR: 14.76; 95% CI: 4.6-46.83; p<0.001), size greater than 5 cm (OR: 1.8; 95% CI: 0.61-6.35; p<0.001), remote metastasis (OR: 34.41; 95% CI: 1.82-115.3; p<0.001), diffuse histological type (OR: 1.43; 95% CI: 0.43-4.71), positive surgical margins (OR: 45.52; 95% CI: 5.88-352.45; p<0.001), non-resectable disease (OR: 54.23; 95% CI: 6.99-420.51), non-adjuvant radiation therapy (OR: 5.87; 95% CI: 1.59-21.65; p<0.001), non-adjuvant chemotherapy (OR: 26.21; 95% CI: 5.79-118.7), and recurrence (OR: 1.62×105; 95% CI: 0.00-35.07×106; p=0.083). 

**Table 5:** Prognostic factors associated with mortality OR: odds ratio; CI: confidence interval; p<0.05

Variable	OR	95% CI	P-value
Depth of invasion (T4)	25.03	6.41-53.54	<0.0001
Tumor size (>5 cm)	1.80	0.61-5.35	<0.0001
Tumor diameter (>5 cm)	5.84	2.32-14.66	<0.0001
Histological type (diffuse)	1.43	0.43-4.71	<0.0001
Distant metastasis	34.41	1.82-116.69	<0.0001
No resected lymph nodes	49.41	6.36-383.55	<0.0001
Nodal status (N3)	14.76	4.6-46.83	<0.0001
Positive surgical margins	45.52	5.88-352.45	<0.0001
Resectable disease	54.23	6.99-420.9	<0.0001
No adjuvant RT	5.87	1.59-21.65	<0.0001
No adjuvant CT	26.21	5.79-118.76	<0.0001
Recurrence	1.02e5	0.00-35.07e6	0.083

The recurrence-related survival predictive factors (Table 6) were T4 injury depth (OR: 14.54; 95% CI: 5.12-41.26; p<0.001), size greater than 5 cm (OR: 5.42; 95% CI: 2.12-13.85; p<0.001), diffuse-type histology (OR: 1.64; 95% CI: 0.49-5.46; p<0.001), remote metastasis (OR: 311.11; 95% CI: 0.62-1.57×105; p<0.001), number of resected nodules (OR: 453.79; 95% CI: 0.9-2.3×105, p<0.001), nodular state N3 (OR: 28.17; 95% CI: 6.18-128.45; p=0.001), positive surgical margins (OR: 42.52; 95% CI: 5.88-128.45; p<0.001), and non-resectable disease (OR: 54.23; 95% CI: 6.99-359.45; p<0.001). Table 7 exemplifies factors related to the disease-free period, T4 depth (OR: 15.57; 95% CI: 5.26-46.10; p<0.001), depth greater than 5 cm (OR: 8.85; 95% CI: 3.42-22.93; p<0.001), diffuse histological type (OR: 1.56; 95% CI: 0.49-4.09, p<0.001), remote metastasis (OR: 482.76; 95% CI: 0.96-2.44×105; p<0.001), number of resected nodules (OR: 940; 95% CI: 1.85-4.77×105; p<0.001), nodular state N3 (OR: 50.47; 95% CI: 10.94-232.79; p<0.001), and positive surgical margins (OR: 612; 95% CI: 1.21-3.1×105; p≤0.001).

**Table 6:** Prognostic factors associated with recurrence OR: odds ratio; CI: confidence interval; p<0.05

Variable	OR	95% CI	P-value
Depth of invasion (T4)	14.54	5.12-41.30	<0.0001
Tumor size (>5 cm)	5.48	2.12-13.86	<0.0001
Tumor diameter (>5 cm)	5.00	1.97-12.67	<0.0001
Histological type (diffuse)	1.84	0.49-5.45	<0.0001
Distant metastasis	311.11	0.62-1.57e5	<0.0001
No resected lymph nodes	463.79	0.00-2.30e5	<0.0001
Nodal status (N3)	28.17	6.18-128.45	<0.0001
Positive surgical margins	45.52	5.88-128.40	<0.0001
Unresectable disease	54.23	6.99-350.40	<0.0001
No adjuvant RT	5.33	1.54-18.50	<0.0001
No adjuvant CT	46.29	5.98-359.42	<0.0001

**Table 7:** Prognostic factors associated with disease-free period OR: odds ratio; CI: confidence interval; p<0.05

Variable	OR	95% CI	P-value
Depth of invasion (T4)	15.57	5.20-46.13	<0.0001
Tumor size (>5 cm)	8.06	3.42-22.80	<0.0001
Tumor diameter (>5 cm)	7.91	3.11-20.13	<0.0001
Histological type (diffuse)	1.58	0.49-4.08	<0.0001
Distant metastasis	482.79	0.96-2.44e5	<0.0001
No resected lymph nodes	840.00	1.85-4.77e5	<0.0001
Nodal status (N3)	50.47	10.94-232.79	<0.0001
Positive surgical margins	812.00	1.21-3.1e3	<0.0001
Unresectable disease	800.00	1.86-4.66e5	<0.0001
No adjuvant RT	4.47	1.35-14.78	<0.0001
No adjuvant CT	35.20	7.71-162.20	<0.0001
Recurrence	27000.00	0.69-5.16e12	<0.0001

## Discussion

Gastric cancer is a global health problem, ranked fourth among recorded neoplasms and third as a cause of cancer death. In recent years, there has been an increase in the incidence of this disease in patients under the age of 45 years, with the current incidence being around 2-15% [1-5].

In Mexico, there are no studies that analyze the relationship between performing R0 maximum effort surgery and survival in this group of patients. The present work analyzed a sample of 104 patients. 

There are several series where they analyze the histopathological characteristics and advanced presentation in these patients, noting a more aggressive presentation of the disease. Among young people, the average age of presentation is around 35 years according to the Lee WJ series. A greater incidence was observed in females compared to males, as reported by the Furukawa series [24].

In relation to the symptoms related to the disease, nausea and dyspepsia were found to be the main symptoms, similar to those obtained in our study. 

With regard to the characteristics of the tumor, the predominant histological type was diffuse. Another relevant characteristic found was the degree of differentiation, with grade 3 standing out, which is related to the aggressiveness of the disease [9]. 

Different series mention advanced disease at the time of diagnosis, with stage IIIA reaching up to 41.3% in our case. Another important result obtained, and undoubtedly the largest predictive factor of survival for gastric cancer, is the nodal status, with 26.9% having 3-6 positive nodes [19].

Oñate-Ocaña and colleagues continuously demonstrated in their study that surgery remains the gold standard in the treatment of gastric cancer. Despite the adverse situations present in our population, the characteristics of the disease limit the treatment. In spite of these adversities, it was possible to carry out R0 resective surgery in 56.7% of cases [34].

An increase in survival of up to 82.9% was observed in patients who were not resected, comparable to Wang series [35-38].

Survival is highly related to the clinicopathological characteristics of the disease at the time of presentation. The factors related to survival include the nodal status, with the best prognosis being the absence of positive nodes, and a surgery that guarantees the complete resection of the disease with adequate nodal dissection, preferably a type D2. Additionally, the absence of distant metastatic disease is also important for a favorable prognosis.

## Conclusions

Gastric cancer is more common in older adults, but the incidence in young adults under 45 years old has been increasing. These young gastric cancer patients often present with more aggressive tumor behavior, including more advanced stage at diagnosis, higher prevalence of poor differentiation and signet ring cell carcinoma, and greater likelihood of peritoneal metastasis. Young gastric cancer patients can have similar or even better overall survival compared to older patients, especially in the resectable setting. While predictive factors for survival and recurrence do not seem to differ by age, maximizing the surgical effort to achieve complete (R0) resection appears to be a key factor in improving outcomes for young gastric cancer patients. Further research is still needed to fully characterize the unique biology and optimal management of gastric cancer in young adults.
